# Retraction Note: Perceptions and experience of rural older people in oral health management in China: a qualitative study

**DOI:** 10.1186/s12903-026-09262-x

**Published:** 2026-07-21

**Authors:** Ran An, Guanghua Jiang, Zitong Wu, Meizi Liu, Muhammad Sohaib, Wenfeng Chen

**Affiliations:** 1 Teaching and Research Section of Clinical Nursing, Xiangya Hospital Central South University, No.87 Xiangya Road, Changsha, Hunan Province China; 2 Xiangya School of Nursing, Central South University, Changsha, China; 3 College of Nursing, Henan University of Traditional Chinese Medicine, Zhengzhou, China

**Retraction Note: BMC Oral Health 24, 644 (2024)**

**https://doi.org/10.1186/s12903-024-04401-8**

The Editor has retracted this article.

Following publication, it was brought to our attention that there is substantial text overlap with a previously published article in Chinese [1].

The authors have not responded to correspondence regarding this retraction.
